# The Effect of Drag and Attachment Site of External Tags on Swimming Eels: Experimental Quantification and Evaluation Tool

**DOI:** 10.1371/journal.pone.0112280

**Published:** 2014-11-19

**Authors:** Christian Tudorache, Erik Burgerhout, Sebastiaan Brittijn, Guido van den Thillart

**Affiliations:** 1 Institute Biology Leiden, Leiden University, Leiden, The Netherlands; 2 NewCatch B.V., Leiden, The Netherlands; Institut National de la Recherche Agronomique (INRA), France

## Abstract

Telemetry studies on aquatic animals often use external tags to monitor migration patterns and help to inform conservation effort. However, external tags are known to impair swimming energetics dramatically in a variety of species, including the endangered European eel. Due to their high swimming efficiency, anguilliform swimmers are very susceptibility for added drag. Using an integration of swimming physiology, behaviour and kinematics, we investigated the effect of additional drag and site of externally attached tags on swimming mode and costs. The results show a significant effect of a) attachment site and b) drag on multiple energetic parameters, such as Cost Of Transport (COT), critical swimming speed (U_crit_) and optimal swimming speed (U_opt_), possibly due to changes in swimming kinematics. Attachment at 0.125 bl from the tip of the snout is a better choice than at the Centre Of Mass (0.35 bl), as it is the case in current telemetry studies. Quantification of added drag effect on COT and U_crit_ show a (limited) correlation, suggesting that the U_crit_ test can be used for evaluating external tags for telemetry studies until a certain threshold value. U_opt_ is not affected by added drag, validating previous findings of telemetry studies. The integrative methodology and the evaluation tool presented here can be used for the design of new studies using external telemetry tags, and the (re-) evaluation of relevant studies on anguilliform swimmers.

## Introduction

Telemetry studies on aquatic animals often use external tags to monitor temporal and spatial movements and answer ecological questions. However, external tags are known to impair swimming energetics dramatically in a variety of species including penguins, seals, turtles and dolphins, through added drag [1]–[5] and reduced manoeuvrability [6]–[8]. Similarly, various eel species were tracked during their open ocean migration, using externally attached “pop-up satellite archival tags” (PSAT; e.g. *Anguilla. dieffenbachii* [9], *A. japonica* [10], various tropical eels [11]) including the critically endangered [12] European eel (*A. anguilla*, [13]). Also in this species, it has been shown that PSATs increase the Cost Of Transport (COT) up to 3-fold [14], [15] and possibly impair escape manoeuvres from predators [16].

Compared to other aquatic animals, eels have an extremely high swimming efficiency [17]–[20], up to six times higher than rainbow trout [21] and some 1.3–1.4 times the values for other species [22]. This high swimming efficiency is possibly based on a combination of low drag and high thrust of the anguilliform swimming mode [23], therefore making them susceptible to added drag.

PSATs were previously attached near the Centre Of Mass (COM; [9], [13], [14]. However, the COM of a swimming eel varies in position and lies regularly outside of the body due to actively oscillation with lateral wave movements [24], [25]. A tag at that position could therefore not only increase the drag, but also may impair the equilibrium, manoeuvrability and escape behaviour.

In order to study the long distance migration of eels, and therefore to contribute to their efficient protection worldwide through informing conservation effort on their migration behaviour, telemetry studies are inevitable. However, it appears that added drag through external tags impairs swimming energetics and behaviour, especially interfering with the highly efficient anguilliform swimming mode. Eels are therefore a sensitive model to experimentally study the effect of external tags on swimming energetics and kinematics. Additionally, predictions based on theoretical models alone will misestimate the effect on freely moving organisms [26].

Using spherical shaped drag dummies, since the drag force of a sphere depends less on surface friction than on the shape drag [27], the present study aimed to a) evaluate the effect of the attachment site, b) quantify the effect of added drag on eel swimming performance, and c) identify methods for determining the effect of added dag of external tags for the use in telemetry studies, evaluation of existing data, and the design of novel tags.

## Materials and Methods

### Animals and housing

Farmed female silver eels (N = 8; body weight, bw: 649.4±131.2 g; body length, bl: 657±42 mm; maximum cross sectional area: 150.5±9.6 mm^2^. All values are mean ± standard error (SE); silver index 3–4 [28]; origin: Passie voor Vis B.V., Sevenum, The Netherlands) were used since they show a lower susceptibility to handling stress and a lower variety in physiological response than wild eels, but similar swimming performance and swimming fitness values [20]. After transport to the laboratory in early May, eels were acclimated for ca. two months in a 7000 L recirculation system, supplied with natural seawater (28±1 ppt) at 18±1°C with an air saturation of 75–85% in a density of 14 fish per volume (of which 8 were used). Light was dimmed before and during the trials to reduce stress. As the eels cease feeding when silvering, they were not fed during the whole period of time. The eels kept their silver stage during the entire experimental period.

### Attachment-site and support device

**Figure 1:**
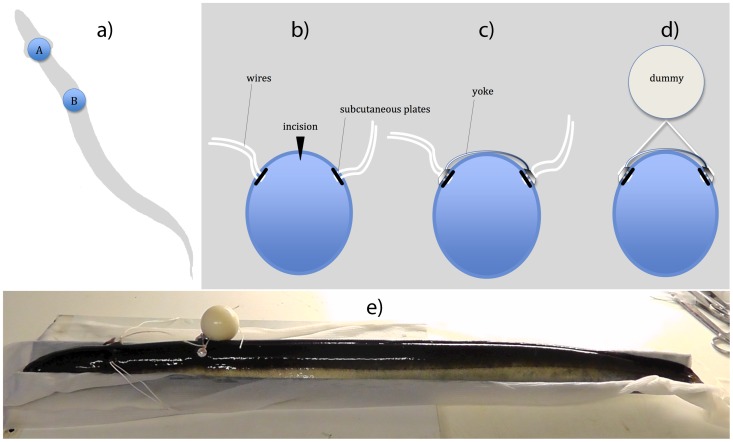
Attachment site and device. a) Diagram of attachment sites A and B. Site A represents a part of the body with minimal oscillation movement during swimming (0.125bl from the tip of the snout), while site B represents the Centre Of Mass, used in previous studies as attachment site (0.35bl). b - d) Diagram of attachment device on the eel: b) Two Teflon plates are inserted through a central dorsal incision and positioned ca 2.5 cm lateral of the incision under the skin with a braided silk thread conducted through the skin. c) A custom made “yoke” (transport device) is secured tightly with the thread on the skin of the eel. d) The threads were further connected with the drag dummies. e) Photography of an eel on the surgery table with silk threads at site A and an attached intermediate dummy at site B.

In order to test the effect of attachment-site on swimming energetics and kinematics, the following two sites were chosen (fig. 1a):

site A) 0.125 bl from the tip of the snout, the most posterior site of minimal lateral body movement [25].
site B) 0.35 bl (COM) from the tip of the snout, approximately corresponding with the attachment site in previous studies [9], [13], [14].

**Figure 2:**
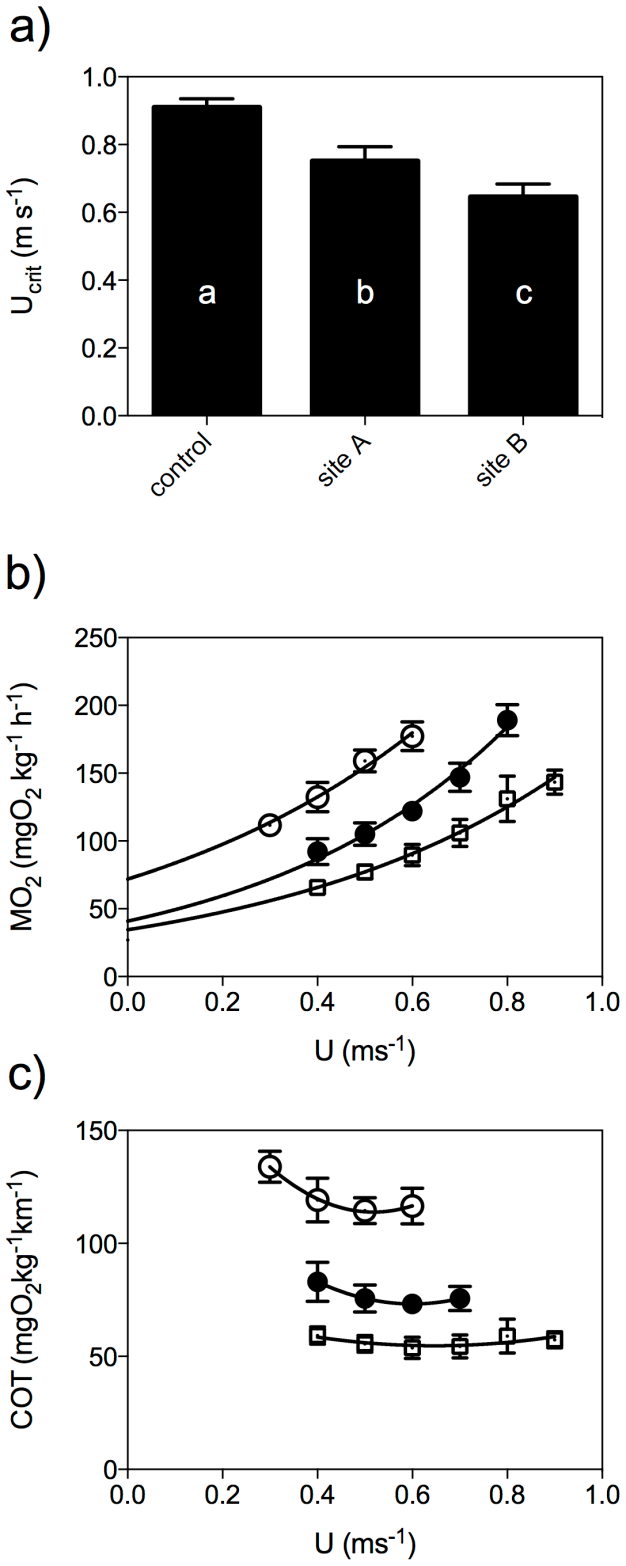
Swimming energetic parameters with increasing drag. a) Critical swimming speed (U_crit_), b) relative oxygen consumption (MO_2_, mgO_2_kg^−1^h^−1^) and c) Cost Of Transport (COT, mgO_2_kg^−1^m^−1^), both as a function of swimming speed (U, m s^−1^) for the first (□) and second control (⋄), and for eels carrying the support device (▵), a small (•), intermediate (•) and a large (•) dummy at site A. Exponential function MO_2_  =  SMRe^cU^, with SMR the standard metabolic rate, e Euler's constant and c constant, and U swimming speed (for values see table 1). Data are mean±SE, repeated measures ANOVA, p<0.05, N = 8, r^2^>0.9, * indicates significant difference from control at the respective speed.

The support device consisted of two parts: 1) the subcutaneously implanted Teflon plates (Ø 7 mm×1.2 mm depth) to reduce shear stress on the skin, equipped with a thread (1 mm diameter) of braided suture silk (OEM, Shanghai, China) through two holes, 2 mm apart (fig. 1b), and 2) a “yoke” which had the function to hold the plates in position (fig. 1c). The yoke consisted of two Teflon plates (Ø 7 mm×2 mm depth) with two holes, 2 mm apart of each other, which were connected by a slightly bend stainless steel wire. This yoke was custom made for each eel and secured with the silk threads conducted through the holes. The loose ends of the threads could be tied to a tag dummy (fig. 1d), which was situated at a distance of ca. 2 cm from the body.

### Drag dummies

The drag dummies (hereafter named dummies) were made from acrylonitrile butadiene styrene (ABS), which is neutrally buoyant in seawater (density ca. 1040 kg m^−3^), and spherical in shape. As the drag force of a sphere depends less on surface friction and more on the shape drag [27], it can be calculated from the diameter of the sphere according to the formula 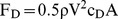
with F_D_ the drag force in N, ρ the mass density of the fluid (1020 kg m^−3^), V the water velocity in m s^−1^, c_D_ the drag coefficient (0.45 for a sphere), and A the diameter in m [54]. V corresponded approximately to the optimal swimming speed (U_opt_), the swimming speed with the minimum Cost Of Transport (COT_min_), of the first control group (i.e. 0.65 m s^−1^, see Results section), and A of the spheres resulted in 2.56 cm for 0.05 N (hereafter named ‘small’), 3.62 cm for 0.1 N (hereafter named ‘intermediate’) and 5.13 cm for 0.2 N (hereafter named ‘large’). In order to confirm the calculated values, the drag force was measured separately with a force transducer (Correx, Switzerland) in a swimming tunnel at water speeds of 0.2 to 0.9 m s^−1^ in intervals of 0.1 m s^−1^ in triplo. Averages of the measured values were expressed as a polynomial function of water velocity (V, m s^−1^). This resulted in the following functions for the different dummy sizes: F_D_ = 0.123V^2^–0.004V for the small, F_D_ = 0.341V^2^–0.065V for the intermediate, and F_D_ = 0.56V^2^–0.068V for the large dummy (r^2^>0.99). The calculated and measured values did not differ for more than 5%.

### Surgery and handling

Eels were anesthetised with clove oil (1∶10 dissolved in 96% ethanol, 1 ml in 1 l seawater [15]). When fully immobile after maximum 10 minutes of anaesthesia, they were placed on the operation table on a half cylindrical support covered with a wet towel. Surgery or attachment procedures lasted under one minute (30–60 s). If eels moved during handling, they were reintroduced into the anaesthesia bath for a short period. For positioning the teflon plates subcutaneously (fig. 1b), an incision of ca. 5 mm was made dorsally at the two sites described above (A and B). The two round plates, equipped with a silk thread, were inserted and pushed gently into position under the skin, ca. 15–20 mm right and left of the incision. The threads were conducted through the skin outwards using two surgical needles, and the incision was subsequently closed using cyanoacrylate glue (Loctite, Düsseldorf, Germany). After surgery, eels were released in the holding tank, where they recovered from anaesthesia within 5 to 10 minutes. Finally, eels were allowed to recover in the holding facility for at least five days.

Before placing the eels into the tunnels, they were anaesthetised and the yoke was attached by conducting the thread through the holes and tied into a knot, fixing the yoke tightly to the skin of the eel as described above (fig. 1c). A dummy could be attached to the eel by knotting it to the threads. A distance of 2 cm was maintained between the body of the eel, and the lower edge of the dummy (fig. 1d). A picture of an eel on the surgery table is added in figure 1e.

### Swimming trial sequence

Seven swimming trials, consisting of combined swimming energetics and kinematics tests, were conducted on 8 individual eels. These trials were completed in the following sequence on consecutive periods of two days.

1. Control 1: eels swam without a tag or a support device, for the establishment of baseline values (see below),
2. Support device: eels swam with a support device only, attached at site B, which was considered to be a more impairing site, to test for handling effects.
3. Eels swam with an intermediate dummy attached at site B,
4. Eels swam with an intermediate dummy at site A; these steps established the comparison for attachment site, with site A being less impairing than site B. Therefore experiments were continued with attachment at site A
5. Eels swam with a small dummy at site A
6. Eels swam with a large dummy at site A

For trial 5 and 6, the dummy sizes were assigned alternatingly, so half of the eels swam with an intermediate, small and then large dummy and the other half swam with an intermediate, large and then small dummy, to avoid a habituation effect.

1. Control 2: eels swam without a tag or support device, to test for habituation effects.

Since the animals remained in the swimming tunnels during the entire course of the experiment, with the exception of the times when they were handled for surgery, attachment and detachment of dummies etc., individual marking (e.g. by pit tagging) was not necessary.

### Swimming energetics

Eels were anesthetized for preparatory handling before each swimming trial i.e. measurement of body weight and length (for identification and monitoring of well being), or attachment, or detachment of a support device and/or a dummy, as described earlier. Subsequently, the eels were transferred to a 127L Blazka-type swimming tunnel [18] connected to the recirculation system of the holding facility with the same water conditions, where they were allowed to recover for 16 to 24 hours at a resting velocity of 0.1 m s^−1^ to keep the water well oxygenated.

After recovery, the animals were subjected to a critical swimming speed (U_crit_) test. Water velocity was increased in increments of 0.1 m s^−1^ at intervals of 20 min [14] until the fish fatigued, i.e. refused to swim and was flushed against the downstream grid of the tunnel. After fatigue, fish were allowed to rest at a water speed of 0.1 m s^−1^.

U_crit_ was calculated according to the equation: 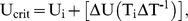
where U_i_ is the highest velocity maintained for the entire 20 min interval, ΔU is the velocity increment (0.1 m s^−1^), T_i_ is the duration of the final (fatigue) step and ΔT is the time interval (20 min; [29]).

After recovery of 16 to 24 hours, eels were subjected to a series of swimming speeds ranging from 0.3–0.9 m s^−1^ with increments of 0.1 m s^−1^ and 60 min intervals, for the determination of oxygen consumption rate, which was measured during the last 30 min of each swimming period, with a significant slope in the [O_2_] decline (p<0.05, r^2^ = 85.7±2.5). Subsequently, the tunnels were flushed with oxygenated water from the holding system for a period of 30 min (air saturation 85.4±3.6%).

Mass specific oxygen consumption (MO_2_ in mgO_2_ kg^−1^ h^−1^) as a function of swimming speed (U) was fitted to the exponential equation [30]: 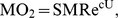
with SMR being the standard metabolic rate and e being Euler's constant and c being a constant. The SMR was extrapolated mathematically to zero swimming speed [14]. U_opt_, the optimal swimming speed (m s^−1^), i.e. the swimming speed with the minimum Cost Of Transport (COT_min_), was calculated from this exponential function by
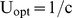
and COT_min_, i.e. the swimming costs per distance swum at U_opt_ (in mgO_2_ kg^−1^ km^−1^), was calculated by 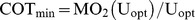

[31]. For large dummies, only one data point was available at 0.6 m s^−1^, which was incorporated in the calculation.

Resulting swimming speeds (U_crit_, U_opt_) and resulting calculations were corrected for the solid blocking effect according to [32]: 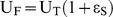
with U_F_ the corrected speed, U_T_ the original speed, and ε_S_ the fractional error quotient: 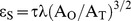
with τ a dimensionless factor depending on flume cross-sectional shape (0.8), λ a shape factor for the test object (0.5), A_O_ the maximum cross-sectional area of the fish, and A_T_ the cross-sectional area of swimming section.

### Swimming kinematics

A HD video camera (30 frames per second, Panasonic, HDC-SD90, Panasonic Inc., Japan) was mounted 0.6 m above the swimming section. To compensate for the spherical aberration created by the cylindrical swimming tunnel, a Perspex adapter box with a flat surface and a concave underside, filled with water, was fitted tightly on top of the tunnel. The eels were filmed for 20 min at each speed (range 0.3–0.9 m s^−1^). Per swimming speed, 3 movie sequences of 20 s (randomly chosen as described by [33]) from the beginning, middle and last part of the 20 min video recordings, were used for further analysis. In short, the period of 20 min was divided in three periods of 400 s, which was then divided in 20 periods of 20 s. One period of 20 s was then chosen using a mathematical randomisation function (Microsoft Excel:Mac 2011, Microsoft Inc., Seattle, USA). From each section of 20 s of in total 9 resulting measurements per swimming speed, tail beat frequency (f), amplitude at the tip of the tail, site A and B (a, a_A_, and a_B_, resp.) and body wave velocity (W) were measured: f was obtained by counting during the entire period of 20 s, amplitudes were calculated as the difference between two outermost positions, and W was calculated as the distance travelled by a wave crest over time, using Vernier Logger Pro (v3.6., Vernier Software & Technology, USA). The dimensionless Strouhal number (St) has been shown to be strongly correlated to force production and efficiency of flapping foils [34] and to the propulsive efficiency of swimming fish [35], [36], and was calculated as St = a.f/U [25].

## Statistics

Data and residuals were tested for normal distribution by Kolmogorov-Smirnoff test; after confirmation (p<0.05, N = 8), data of different experimental treatments were compared using repeated measurements ANOVA followed by a Holm-Sidak multi comparison procedure (SigmaPlot v. 11, Systat systems inc. USA) when significant effects were found. Significance value was p<0.05. Data are given as mean ± SE.

### Ethics Statement

This study complied with the Dutch Law on Animal Experiments and were approved by the Animal Ethical Committee of Leiden University (DEC# 10231). All surgery was performed under clove oil anaesthesia, and all efforts were made to minimize suffering and reduce the number of animals used.

## Results

### Surgery and handling

Eels were completely unresponsive under anaesthesia, and subsequently responded well to anaesthesia, surgery, attachment of the dummies, and handling, with no mortalities and no infections observed over the entire course of the experiments (2.5 months). After release in the holding tank or the swimming tunnels, they recovered after 5 to 10 min, showing routine activity, sometimes resting at the back of the tunnel. There was no avoidance behaviour against the attached dummies, such as scratching, rubbing or probing of the attachments, or the affected part of the body with mouth or tail.

### Swimming behaviour

At low water velocities eels would remain coiled up against the rear grid of the swimming tunnels. At water speeds of 0.4 m s^−1^ and above, control animals would orient themselves against the stream and hold position in the tunnel using a regular swimming mode, characterised by a steady anterior position, visually uniform tail beat frequency and amplitude. However, animals equipped with a large dummy at site A or an intermediate dummy at site B, positioned themselves against the stream and swum already at velocities of 0.3 m s^−1^, but irregularly, defined as unsteady position, frequent acceleration and deceleration during the velocity periods, often in contact with the rear grid of the tunnel. However, this irregular swimming mode did not persist at velocities from velocities of 0.4 m s^−1^ onwards. Also, these animals showed a slight rotational movement from side to side, correlated with the tail beat frequency. This rotational movement was not observed with control animals and animals equipped with small or intermediate dummies at site A.

### Attachments site

In order to test the effect of attachment-site, intermediate dummies were attached at site A or site B, and swimming energetics and kinematics were compared between each other and to control 1. Analysis of energetic values revealed that critical swimming speed (U_crit_) for site B was significantly lower (ca. 15%) than for site A (p<0.05, N = 8), with both lower (ca. 30 and 15%, respectively) than control 1 (p<0.05, N = 8, fig. 2a). Oxygen consumption rates (MO_2_) for site A were significantly higher than control values at speeds of 0.6 m s^−1^ and above, for site B higher than for site A and control 1 at all speeds (fig. 2b). The extrapolated standard metabolic rate (SMR) for site A did not differ significantly from control 1, but was significantly elevated for site B (p<0.001, N = 8; fig. 2b, table 1). The Cost Of Transport (COT) values for site A were significantly higher than control values at all speeds, as they were for site B compared to site A and control values (p<0.05, N = 8; fig. 2c). Finally, minimum Cost Of Transport (COT_min_, table 1) was significantly higher for site B compared to site A, which in turn was higher than for control 1 (p<0.001, N = 8). The optimal swimming speeds (U_opt_) did not differ between attachment sites (table 1). Kinematic values show that both, tail beat frequency (f) and body wave velocity (W), plotted against swimming speed (U), revealed a linear relationship, i.e. f = a+bU, and W = a+bU, with a the intercept and b the slope (table 2). Analysis reveals that f, W and the related b values were higher when a dummy was attached at site B compared at site A, and both compared to control 1 (p<0.05, N = 8, table 2). Tail beat amplitude at the tail tip (a) or the different attachment sites (a_A_ and a_B_) did not differ significantly (table 2). The Strouhal number was significantly higher for site B compared to site A (p<0.05, N = 8; table 2). These results indicate an effect of the attachment site on both energetic and kinematic parameters, with site B being more disadvantageous than site A.

**Table 1:** Swimming energetics.

	control 1	tag support B	small A	intermediate A	intermediate B	large A	control 2
SMR(mgO_2_ kg^−1^ h^−1^)	40.58±6.45^a^	36.85±1.95^ a^	42.10±2.41^a^	42.55±7.43^a^	65.88±10.76^b^	79.65±15.38^c^	36.96±2.45^a^
U_opt_(m s^−1^)	0.68±0.03^a^	0.69±0.02^a^	0.69±0.03^a^	0.65±0.12^a^	0.61±0.11^a^	0.47±0.09^b^	0.70±0.08^a^
COT_min_(mgO_2_kg^−1^ km^−1^)	54.79±9.74^a^	54.59±3.33 ^a^	62.87±3.11^a^	68.83±12.09^b^	111.76±10.68^c^	138.94±29.4^d^	54.82±3.01^a^

Oxygen consumption (MO_2_, mgO_2_ kg^−1^ h^−1^) expressed as a function of swimming speed (U, m s^−1^) as MO_2_  =  SMRe^cU^, with SMR the standard metabolic rate, e Euler's constant, and c contant, and the derived optimal swimming speed (U_opt_, m s^−1^) and minimum Cost Of Transport (COT_min_, mgO_2_ kg^−1^ km^−1^). Values are mean ± SE, letters indicate significant differences per row, repeated measurements ANOVA, p<0.05, N = 8. Control 1, tag support, small dummy attached at site A (fig.1a), intermediate dummy at site A and at site B (fig.1a), large dummy at site A, and control 2.

**Table 2:** Swimming kinematics.

	control 1	tag support B	small A	intermediate A	intermediate B	large A	control 2
W intercept	0.23±0.05	0.26±0.11	0.24±0.07	0.24±0.10	0.45±0.12*	0.35±0.10*	0.26±0.04
W slope	1.05±0.11	1.00±0.11	1.05±0.12	1.12±0.18	1.65±1.67*	2.03±0.27*	1.02±0.08*
f intercept	1.26±0.14	1.29±0.13	1.32±0.2	0.52±0.26*	0.68±0.48*	0.48±0.49*	1.32±0.28
f slope	2.15±0.16	2.14±0.12	2.09±0.23	2.45±0.77*	4.98±1.67*	5.98±0.82*	2.08±0.32
a (cm)	7.6±1.6	7.8±1.7	7.8±1.6	7.8±1.7	7.9±3.6	8.2±3.8	7.5±1.7
a_A_(cm)	0.8±0.2	0.8±0.2	0.8±0.3	0.9±0.2	0.9±0.5	0.9±0.6	0.8±0.3
a_B_(cm)	2.5±1.2	2.5±1.1	2.5±1.2	2.6±1.2	2.7±1.4	2.7±1.2	2.5±1.1
Strouhal number	0.32±0.12	0.33±0.07	0.31±0.08	0.39±0.04	0.74±0.12*	0.83±0.04*	0.32±0.07

Body wave velocity (W, m s^−1^) and tail beat frequency (f, Hz), correlate linearly with swimming speed (U, m s^−1^) i.e. f = a+bU or V = a+bU, with intercept (a) and slope (b); amplitude at the tail tip, site A and B (a, a_A_ and a_B_; cm); Stouhal number (dimensionless). Values are mean±SE, * indicate significant differences from control1 (repeated measurements ANOVA, p = 0.05, N = 8). Control 1, tag support only, small dummy attached at site A (fig.1a), intermediate dummy at site A and at site B (fig.1a), large dummy at site A, and control2.

### Effect of additional drag

**Figure 3:**
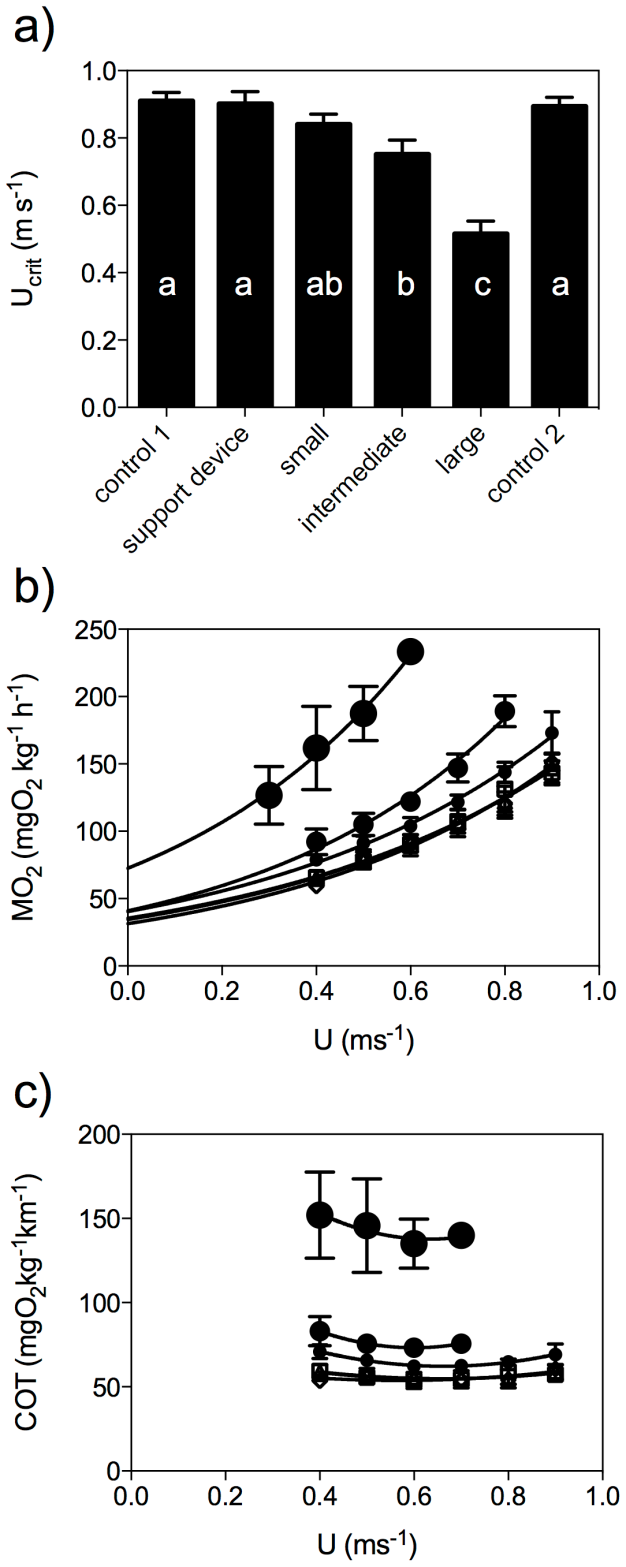
Swimming energetic parameters with attachment site. a) Critical swimming speed (U_crit_), b) relative oxygen consumption (MO_2_, mgO_2_kg^−1^h^−1^) and c) Cost Of Transport (COT, mgO_2_kg^−1^m^−1^), both as a function of swimming speed (U, m s^−1^) for control (□), and for eels carrying an intermediate dummy at site A (•) and B (○). Exponential function MO_2_  =  SMRe^cU^, with SMR the standard metabolic rate, e Euler's constant and c constant, and U swimming speed (for values see table 1). Data are mean±SE, repeated measures ANOVA, p<0.05, N = 8, r^2^>0.9, * indicates significant difference from control at the respective speed.

In order to test the effect of additional drag force (F_D_ at U_opt_ of control 1) on swimming energetics and kinematics, small, intermediate and large dummies (F_D_ = 0.05 N, 0.1 N and 0.2 N respectively) were attached at site A, since this site showed to be less disturbing for attachment than site B, and compared to control 1. Small dummies did not significantly affect U_crit_ (p>0.05, N = 8), only intermediate and large dummies reduced U_crit_ significantly (p<0.05, N = 8; fig. 3a). Small dummies did not significantly affect MO_2_ values, only MO_2_ values for intermediate dummies at speeds of 0.6 m s^−1^ and higher and for large dummies at all speeds, were significantly higher than for control 1 (fig. 3b). Only large dummies led to a significantly higher SMR value (p<0.001, N = 8, fig. 3b, table 1). Small dummies did not significantly affect COT values, but intermediate and large dummies led to a significant increase in COT values when compared to control 1 at all speeds (p<0.05, N = 8; fig. 3c). Only one fish carrying a large dummy was able to swim at 0.6 m s^−1^. Large dummies led to a significant decrease of U_opt_ and an increase of COT_min_ (p<0.05, N = 8; table 1). Finally, intermediate and large dummies led to an increase in fin beat frequency (f) and body wave velocity (W) at speeds greater than 0.4 m s^−1^, with a significant increase in slope (p<0.05, N = 8; table 2). Tail beat and body point amplitudes a, a_A_ and a_B_ did not differ significantly between treatments. The Strouhal number was only significantly increased with a large dummy (p<0.05, N = 8; table 2). These findings indicate an effect of added drag on swimming energetics and kinematics.

### Training effect and support device

To estimate a possible training effect due to repeated testing, or a handling effect of attaching the support device, control group 1 was compared to a group carrying a support device only at site B, and to a control group with removed support device, at the end of the trials (control 2). There were no significant differences between energetic values (fig. 3a, fig. 3b and table 1) or between kinematic values of the different treatments. Therefore, these results indicate no effect due to training or handling during the trials.

### Comparison of U_crit_ with COT_min_

**Figure 4:**
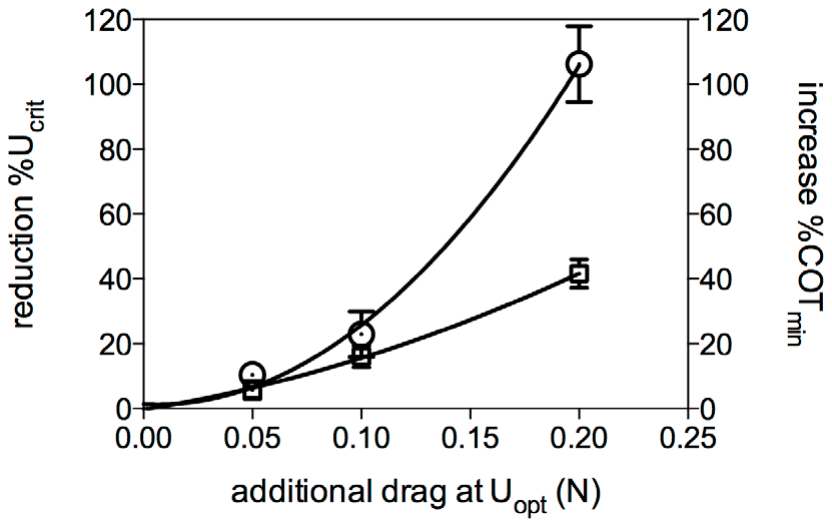
Relation of critical swimming speed (U_crit_) and minimum Cost Of Transport (COT_min_) with added drag. Percentual decrease of U_crit_ (reduction %U_crit_; □) and percentual increase of COT_min_ (increase %COT_min_; ○) per individual, plotted against additional drag force (F_D_, N, measured at U_opt_, 0.68 m s^−1^) of small (F_D_ = 0.05 N), intermediate (F_D_ = 0.10 N) and large dummies (F_D_ = 0.20 N) at site A. The resulting polynomial graph with best fit (r^2^>0.99) followed the formula red%U_crit_ = 102.8±49.35F_D_^2^+527.7±273.8 F_D_ and inc%COT_min_ = −8.9±113.0F_D_^2^+2687±636.7F_D_, respectively.

For the evaluation of past and future telemetry studies using external tags, corresponding changes in U_crit_ and COT_min_ values were compared and modelled. By plotting the effect of the dummies (E) as a) the reduction of % U_crit_ per individual (red%U_crit_) or as b) the increase of % COT_min_ per individual (increase%COT_min_), over the different drag values (F_D_), the resulting polynomial curve (fig. 4) shows an increase following the formula E = aF_D_+bF_D_^2^ with a and b being constants. The values of a and b are for red%U_crit_ 102.8±49.35 and 527.7±273.8, and for increase%COT_min_ −8.92±113 and 2687±626.7, respectively. The values for increase%COT_min_ and red%U_crit_ at 0.05 and 0.1 N F_D_ did not differ; however the values at 0.2 N differed significantly from each other (p<0.05, N = 8). These results provide a practical tool for the evaluation of comparative methods for the estimate of the effect of added drag on energetic parameters.

## Discussion

The European eel is a species typical for the waters of Western Europe. The spawning site of this fascinating species is still a mystery, however. The Danish biologist Johannes Schmidt found the smallest eel larvae (leptocephalus) in the Sargasso Sea, strongly indicating a spawning site [37], [38], [39]. Therefore the maturating silver eels must cross more than 6000 km partly on the sea bottom at pressures of 200 atmospheres and without feeding [40]. Still, an adult spawning eel has never been observed in the Sargasso Sea, nor were eggs found. Assuming a cruising speed of 0.8 to 1 BL/sec [20] eels would perform the 6000 km journey in 4 to 6 months. However, the Icelandic and Moroccan eels might belong to different populations, suggesting spatially or temporally separated spawning groups [41]. These facts add to the fascination for this highly endangered species and the urgency for its protection [12]. The aim of the present study was to help improve tagging methods in order to successfully follow this species on its spawning migration and inform conservation effort. Therefore we quantified the drag of external tags on the highly efficient anguilliform swimming mode, integrating swimming physiology, behaviour and kinematics. The results show that a) attachment site and b) relatively low added drag have significant effects on a variety of swimming parameters, possibly due to the extreme efficiency of anguilliform swimming. These results can help to design new telemetry devices, outline new studies and re-evaluate existing telemetry data on eels and other aquatic species.

### Surgery technique and tag support

Previous studies used nylon wires, conducted through skin and deep muscle layer, to hold the tag or dummy into place [9], [13], [14]. This method, however, could affect swimming capacity, motility and behaviour. The present attachment method to the skin is assumably less invasive, with the yoke keeping the tag in position, and therefore reducing lateral oscillation of the dummy. Additionally, the Teflon discs may spread the strain over the skin and did not result in additional damage during swimming, which corroborates with the observation of Økland et al. [42], who also suggest an attachment method using the skin, since eel skin has a high sheer strength and can endure forces of 40–60 MN m^−2^. We therefore suggest an attachment method on the skin instead of through the muscle layer.

### Attachment site

Attachment near the Centre Of Mass (COM; site B, fig 1a), as compared to the most posterior site of minimal lateral body movement (A; fig 1a), reduced critical swimming speed (U_crit_) and increased relative oxygen uptake (MO_2_), minimum Cost Of Transport (COT_min_) and standard metabolic rate (SMR), and it impaired kinematic parameters (tail beat frequency f, Strouhal number St, body wave velocity W). Additionally, a sagittal rotational movement was observed, possibly compensating for the inertia force pivoting above the Centre Of Mass, also previously reported by Webb [26]. This force is proportional to the amplitude, which is larger at site B than at site A (table 2). In anguilliform swimmers, the Centre Of Mass is an actively moving part of the body, used for propulsion by means of horizontal oscillation [25], and is therefore not suited for external tagging. An increase in the extrapolated Standard Metabolic Rate (SMR, table 1) suggests an increased stress response [43]. Based on our results, it can be concluded that site A is the better choice for external tags.

### Added drag

The results show that added drag significantly impairs swimming parameters such as U_crit_, MO_2_ and COT_mint_. The SMR was increased by additional drag force (F_D_) of 0.2 N. Kinematic parameters (f, St and W) were also negatively affected by added drag. These results reflect the findings of other previous studies concerning alteration in overall drag [26, 44, 15. 14].

The U_crit_ test was criticised in the past for its susceptibility to experimental factors [45], [46], [47]. Nevertheless, U_crit_ is valid in a comparative study such as the present. Because U_crit_ values reflect aerobic as well as anaerobic powered swimming capacity [29], [48], the U_crit_ test is a first evaluation of external tags [44]. However, when comparing the relative alteration of U_crit_ with that of COT_min_ due to added drag (fig. 4), it appears that the effect on U_crit_ corresponds to the effect on COT_min_ only up to 0.1N F_D_. This limits the use of U_crit_ tests for estimating the effect of added drag on swimming capacity in the field. It is therefore recommended to estimate the limitations of U_crit_ tests on a species base. Interestingly, at values higher than 0.1N, the effects on COT_min_ are increasingly higher than on U_crit_, indicating additional compensation by e.g. anaerobic metabolism, as suggested by Webb [26]. Future research will elucidate the effect of added drag on the anaerobic metabolism, by measuring volitional sprint speeds and times [49].

Added drag up to 0.1 N did not reduce U_opt_, a result previously found by Methling et al. [14]. Possibly, migrating with a tag at a reduced U_opt_ would minimize the Cost Of Transport. But it would also prolong the journey. Even with similar U_opt_, COT_min_ was significantly higher for the animals with added drag of up to 0.1N, possibly for synchronising the arrival at the spawning grounds, regardless the costs. So, while energy expenditure is increased, thereby depleting energy stores more rapidly than would be desirable, swimming speed would be unaffected and all fish, regardless their energetic condition, would reach the spawning site at the same time. In this light, the conclusions regarding swimming speeds of previous telemetry work on eel migration [9], [11], [13] seem well grounded.

### Methodological evaluation

The methodology of this study combines swimming energetics, kinematics and behavioural observation. Similar studies accepted an r^2^>0.9 for oxygen measurements over time [50], [51], while the present results are based on a minimum r^2^ of 0.85. The set up used here is unique and especially designed for anguilliform swimmers. The disadvantage is that a relatively large water volume produces more background noise in the measurements and the r^2^ therefore is reduced. Therefore a higher r^2^ is recommended for future studies. Additionally, previous studies suggest correcting for the solid blocking effect [50], [14], while other studies [52] claim that a correction is not necessary if cross sectional area of the fish is below 10% of that of the swimming tunnel. However, we advise to perform this correction when data are applied to the field, because the actual swimming speed could be significantly greater. Correction for solid blocking effect in the present study led to an increase in water velocities of 4.9±1.2%, which was statistically negligible. Finally, the present study aimed to reduce number of animals (N = 8) and experimental handling, by using repeated measures, testing attachment site using only one tag size (0.1 N), and the support devise at site B only to reduce experimental trials, which is acceptable in a comparative study such as this. Also, the relative effect of added drag is likely to decrease with body size of the eel, but absolute estimates for COT_min_ are variable for reasons other than eel size, such as origin (wild vs farmed [20]) or the infection with swim bladder parasites [53]. Therefore, the described methods to evaluate tagging techniques should be applied in the field on a case-to-case base.

### Recommendations for the use in the field

The results show that even relatively low additional drag can change swimming parameters significantly and the presented model allows the calculation of possible effects of telemetry tags on energetic parameters: commonly used satellite tags by Microwave Telemetry and Wildlife Computers, tested in the study by Grusha & Petterson [3], produce an additional drag of 0.159 N at a water speed of 0.6 m s^−1^, likely more at the reference speed of 0.68 m s^−1^ of the present study. This value lies within the limits of the drag forces tested and would lead to a reduction of U_crit_ by 29.68±14.76% and to an increase of the COT_min_ by 66.51±33.81%. Additionally, being tagged at the traditional site B near the COM, would reduce U_crit_ by additional 15%, resulting in ca. 45% total reduction, and it would increase COT_min_ by additional 63%, resulting in a total increase of ca. 130%. These results, of course, are only valid if we assume an additive effect of added drag and attachment-site. If this effect should be factorial or otherwise related, the resulting effect would be even more dramatic. With other words, being tagged with a commercially available tag at site B, a migrating eel would reach only half its critical swimming speed and swim for nearly one and a half times the costs.

These results confirm the suggestions by previous studies on eels [9], [13] and other aquatic species: In comparison, Adelie penguins (ca 60 cm body length) equipped with a flipper band (ca 0.5 cm width) had a 24% higher COT_min_ [1], large seals (ca 2 m body length) with radio collars (15 cm) experienced an 15% increase in drag force [5] and green turtles (48 cm carapace length) with radio transmitters (14 cm) had a 27% increased COT. These and other studies [3], [4] support the claim that the tolerance for tags should be quantified before tagging studies are carried out, in order to estimate their effect on the data collected.

## Conclusions

The present methodology integrating swimming physiology, behaviour and kinematics appears useful in similar context for a) testing the tolerance of existing constrictions and b) the development of novel tags and for a variety of aquatic animals, as physical or mathematical models alone tend to over- or underestimate the effect of added drag [26]. Since U_opt_ was not affected by the additional drag below 0.2 N, it was concluded that migrating eels choose to consume more energy in order to reach spawning places on time. Other species, however, may pursue strategies to conserve energy and a case-to-case validation of the effect of external tags on different swimming and migration parameters is necessary. Next to energetic values therefore, kinematic and behavioural data can assist to estimate the effect of externally attached tags on survival and reproduction. The integrative methodology and the evaluation tool presented here can be used for the design of new studies using external telemetry tags, and the (re-) evaluation of relevant studies on anguilliform swimmers.
